# Associations Between Microscopic *Balantioides coli* Counts and Precision Dairy-Farming Indicators in Clinically Healthy Dairy Cows

**DOI:** 10.3390/vetsci13101014

**Published:** 2026-09-25

**Authors:** Gabija Lembovičiūtė, Greta Šertvytytė, Justina Krištolaitytė, Karina Džermeikaitė, Ieva Rodaitė, Eimantas Ginkus, Lina Anskienė, Inga Sabeckienė, Samanta Grigė, Akvilė Girdauskaitė, Ramūnas Antanaitis

**Affiliations:** 1 Large Animal Clinic, Veterinary Academy, Lithuanian University of Health Sciences, LT-47181 Kaunas, Lithuania; greta.sertvytyte@lsmu.lt (G.Š.); justina.kristolaityte@lsmu.lt (J.K.); karina.dzermeikaite@lsmu.lt (K.D.); ieva.rodaite@lsmu.lt (I.R.); eimantas.ginkus@lsmu.lt (E.G.); inga.sabeckiene@lsmu.lt (I.S.); samanta.grige1@lsmu.lt (S.G.); akvile.girdauskaite@lsmu.lt (A.G.); ramunas.antanaitis@lsmu.lt (R.A.); 2 Department of Animal Breeding, Faculty of Animal Sciences, Lithuanian University of Health Sciences, LT-47181 Kaunas, Lithuania; lina.anskiene@lsmu.lt

**Keywords:** *Balantioides coli*, dairy, milk lactose, precision dairy farming, fecal microscopy

## Abstract

*Balantioides coli* is an enteric ciliated protozoan detected in cattle, but the association between microscopic *B. coli* counts and precision dairy-farming indicators remains poorly understood. This cross-sectional study evaluated associations between microscopic *B. coli* counts and behavioral, production and milk-composition traits in 83 clinically healthy Holstein cows in parity 2–4 and 20–100 days in milk. Fecal samples were examinated using sedimentation and modified McMaster methods and abundance was expressed as microscopic counts per field of view. Automated farm systems provided eating and rumination times, daily milk yield, milk fat-to-protein ratio, lactose concentration and somatic cell count. Zero-order correlations between microscopic *B. coli* counts and all evaluated indicators were weak and non-significant (r = 0.027–0.190). After adjustment for parity and days in milk, a modest positive correlation was observed with lactose concentration (partial r = 0.296, *p* = 0.007). The remaining adjusted correlations were weak or negligible and statistically non-significant: eating time (r = 0.105), rumination time (r = 0.036), daily milk yield (r = 0.060), milk fat-to-protein ratio (r = 0.064) and log10-transformed somatic cell count (r = 0.015). Overall, microscopic *B. coli* abundance showed limited association with the evaluated indicators, and the lactose finding should be regarded as exploratory.

## Simple Summary

*Balantioides coli* is a zoonotic intestinal protozoan whose biological significance in livestock remains insufficiently understood. Although it may be detected in the feces of apparently healthy cows, it remains unclear whether differences in its abundance are related to feeding behavior, milk production or milk composition. Understanding these relationships may help farmers and veterinarians determine whether higher microscopic *B. coli* counts provide useful information about cow health and productivity or should be interpreted alongside other herd-monitoring indicators. This study examined 83 clinically healthy Holstein cows from a single commercial dairy farm, where all animals were maintained under the same management conditions. Fecal samples were assessed microscopically, while eating and rumination behavior, daily milk yield, milk fat-to-protein ratio, lactose concentration and somatic cell count were obtained from automated farm-monitoring systems. Cows with higher and lower microscopic counts showed no significant differences in rumination time, milk yield, milk fat-to-protein ratio, lactose concentration or somatic cell count. A modest positive relationship with milk lactose concentration was detected only after accounting for lactation number and days in milk. This finding should be considered preliminary and does not demonstrate that the organism directly affects lactose production. Overall, microscopic *B. coli* counts were of limited value as an indicator of impaired productivity or udder inflammation in clinically healthy cows under the conditions of the studied herd.

## 1. Introduction

*Balantioides coli*, previously referred to as *Balantidium coli*, is a ciliated intestinal protozoan capable of colonizing humans and a wide range of domestic and wild mammals [1,2]. Pigs are generally regarded as its main reservoir, although the organism has also been detected in cattle, sheep, goats, horses, camels, non-human primates and other animal species [2,3,4]. Transmission occurs mainly through the fecal–oral route following ingestion of environmentally resistant cysts in contaminated water, feed or other material [5,6]. Within the host *B. coli* has been found to be predominantly located in the large intestine; within this location, the infection has the potential to remain asymptomatic, or, under specific circumstances, it may be accompanied by mucosal invasion, colitis, diarrhea and other gastrointestinal disturbances [5,7,8]. It is becoming increasingly evident that *B. coli* can be transmitted between a variety of hosts within a broad host range and across different environments. This has led to a growing focus on *B. coli* in the context of the One Health approach, which emphasizes the significant role of environmental health in relation to human and animal health [1,9].

The presence of *B. coli* in the feces of cattle does not necessarily indicate the presence of clinically relevant disease. Field investigations have revealed the presence of the organism in both apparently healthy animals and cattle with gastrointestinal infections. However, the prevalence of the organisms varies significantly among different production systems, geographical regions, animal populations and diagnostics methods [9,10]. Moreover, the majority of studies conducted have concentrated on aspects such as prevalence, host range, zoonotic significance and morphological or molecular identification. However, there has been less emphasis on exploring the functional consequences that arise from natural fluctuations in protozoan abundance [9,10,11]. Microscopy remains the most widely used method because it is accessible and permits recognition of characteristic trophozoites and cysts; however, its sensitivity may be affected by sample preservation, parasite concentration, preparation technique and observer experience [12]. Molecular techniques can complement morphological examination by confirming species identity and characterizing genetic variants, although such methods have been applied more extensively in pigs and other reservoir hosts than in dairy cattle [12,13]. As a result, the biological significance of the varying microscopic counts of *B. coli* in clinically healthy dairy cows requires further scientific investigation.

Precision dairy-farming technologies provide an opportunity to investigate whether enteric organisms are associated with subtle changes in animal behavior, productivity or health-related indicators [14]. Sensors integrated into automated milking and herd-management systems can continuously record milk production, feeding activity, rumination, locomotion and other physiological or behavioral variables at the individual cow level [15]. Such systems may identify deviations from animals’ usual patterns before clear clinical signs become apparent, although sensor-derived measurements are influenced by equipment characteristics, management conditions, physiological state and individual variation [15,16]. Rumination time is particularly relevant because it is associated with feed intake, ration composition, rumen function, health status and stage of lactation [17,18]. A meta-analysis of lactating dairy cows demonstrated a relationship between rumination behavior, milk and milk-fat production, ruminal pH, and total-tract digestibility, while also showing considerable variation among diets and experimental conditions [19]. Research has indicated that the correlation between the duration of rumination and quantity of milk produced is not uniform across the various stages of lactation. This finding suggests that behavioral indicators must be interpretated within the physiological context of the cow [20].

Given that *B. coli* may be associated with gastrointestinal disturbances under certain conditions, investigating its relationship with feeding and rumination behavior may provide insight into the possible physiological significance of variations in microscopic counts [5,7,8]. Furthermore, as rumination behavior is associated with digestive function and milk production, evaluating milk-related indicators may help determine whether variation in microscopic *B. coli* counts is accompanied by changes in dairy cow performance [17,18,19].

Milk-based indicators provide complementary information on productivity and physiological status of the cow. Daily milk yield is a direct measure of productive performance, whereas the milk fat-to-protein ratio is commonly used as an indirect indicator of energy balance and metabolic adaptation, particularly during early lactation [21]. Milk lactose is the principal osmotically active carbohydrate in milk and therefore is related to milk volume. Its concentration varies with parity, stage of lactation, mammary function, energy status and udder health [22]. Somatic cell count (SCC) is widely used as an indicator of mammary inflammation and as a screening measure for subclinical mastitis, although it may also vary according to days in milk (DIM) and udder health history [23]. Considered together, milk yield, milk component ratios, lactose concentration and SCC can provide a broader assessment of whether variation in fecal protozoal abundance coincides with detectable productive, metabolic or mammary health changes.

Despite the increasing availability of precision dairy-farming data, published evidence directly examining relationships between fecal microscopic *B. coli* counts and automatically recorded behavioral, production and milk data is very limited. It therefore remains unclear whether higher microscopic counts in clinically healthy cows represent biologically neutral colonization or are accompanied by subtle alterations they may not be evident during routine clinical examination.

The objective of this study was to evaluate the associations between natural variation in microscopic *B. coli* counts and sensor-derived behavioral, milk-production, and milk-composition indicators in clinically healthy Holstein dairy cows. We hypothesized that higher microscopic *B. coli* counts would be associated with differences in feeding and rumination times, daily milk yield, milk fat-to-protein ratio, lactose concentration and SCC, even in the absence of clinically apparent disease.

## 2. Materials and Methods

### 2.1. Ethical Approval

In accordance with the prevailing legislation of the Republic of Lithuania, the present study was conducted in accordance with the Law on Animal Welfare and Protection (Oficialusis savaitraštis Valstybės žinios, 1997, No. 108-2728; 2012, No. 122-6126) and the European Parliament’s Directive 2010/63/EU on the protection of animals used for scientific purposes. Moreover, the present study was conducted in accordance with the European Convention for the Protection of Vertebrate Animals Used for Experimental and Other Scientific Purposes.

All procedures involving animals were reviewed and approved by the Lithuanian State Food and Veterinary Service Ethics Committee (Approval No. G2-298, 5 March 2025). The study was observational in design, and no experimental infection, pharmacological treatment, dietary intervention, feed restriction, or modification of routine herd-management practices was performed. All animal-related procedures were performed in compliance with applicable national and European animal-welfare requirements.

### 2.2. Farm, Animals, and Management Conditions

A cross-sectional observational design was selected to evaluate naturally occurring variation in microscopic *B. coli* counts and its associations with routinely recorded precision dairy-farming indicators under commercial herd conditions. This design allowed the relationship of interest to be examined without experimental manipulation of the animals or alteration of routine management practices. The investigation was carried out on a commercial dairy farm located in the Kaunas region of Lithuania (54.9753923° N, 23.7662303° E) during the period from November to December 2025. The cows were kept permanently indoors in a free-stall housing system and were not provided with access to pasture. Drinking water was available ad libitum. Barn ventilation was regulated using a DeLaval ventilation system (DeLaval International AB, Tumba, Sweden), which maintained air circulation and appropriate environmental conditions within the housing facilities.

Milking was performed using Lely Astronaut^®^ A3 automatic milking units operating under a free-cow-traffic system (Lely Industries N.V., Maassluis, The Netherlands). Individual cow-level production, milk-composition and behavioral data were obtained from the Lely Astronaut^®^ A3 automatic milking system and associated Lely herd-management software. Rumination and eating activity were monitored using neck-mounted Lely Qwes-H-/HR (Lely Industries N.V., Maassluis, The Netherlands) sensors worn by the cows, with the recorded data automatically transferred to the herd-management system. Data were logged at 2 min intervals and transmitted wirelessly to the associated herd-management system [18]. The variables extracted for the present study included cow identification, parity, body weight, DIM, daily milk yield, milking frequency, milk fat and protein concentration, fat-to-protein ratio, lactose concentration, SCC, rumination and eating time, and individual concentrate intake. Rumination and eating times were expressed as minutes per cow per 24 h.

All cows included in the study were managed under the same feeding conditions. A total mixed ration was offered twice daily, during the periods from 06:00 to 10:00 h and from 12:30 to 15:00 h. The ration was formulated for multiparous, high-producing Holstein cows with an approximate body weight of 550–650 kg and an expected daily milk yield of approximately 40 kg. The principal dietary ingredients were grain concentrate mash, lucerne hay, grass silage, sugar beet pulp silage, corn silage, grass hay, wheat straw, and compound feed (Table 1 and Table 2).

Additional concentrate was dispensed individually through the automatic milking system at an approximate amount of 2 kg per cow per day. Feed was moved towards the feeding line 22 times daily using a Lely Juno automated feed-pushing system (Lely Industries N.V., Maassluis, The Netherlands). During the study period, the mean annual milk production of the herd was approximately 12,000 kg per cow. Representative photographs of the investigated farm, including the housing environment, feeding area, and cows equipped with neck-mounted monitoring tags, are presented in Figure 1.

### 2.3. Study Population, Clinical Examination, and Fecal Sample Collection

A total of 107 Holstein cows were initially considered for inclusion in the study. Following clinical examination, 87 cows were classified as clinically healthy. Of these, 4 cows were excluded because they did not meet the predefined lactation number and/or DIM criteria. Accordingly, the sample size was based on the number of cows that met all predefined eligibility criteria within the selected study population. No formal a priori statistical power analysis was performed. The final study population therefore consisted of 83 cows. Eligible cows were in their second, third, or fourth lactation and between 20 and 100 DIM at the time of sampling. Each enrolled cow underwent one clinical examination and provided one fecal sample during the study period.

Before inclusion, all cows were subjected to a routine clinical examination performed by a veterinarian according to the standardized clinical assessment protocol used at the Large Animal Clinic of the Lithuanian University of Health Sciences. The examination included an assessment of the animal’s general condition, behavior, appetite, rectal temperature, rumen activity, fecal consistency, locomotion, and udder health.

Cows were considered clinically healthy when no signs of systemic, gastrointestinal, metabolic, reproductive, locomotor, or udder disease were detected. Animals showing an abnormal general condition or any other clinically apparent disorder were excluded from the study. Only cows producing normally formed feces and showing no clinical evidence of gastrointestinal disease were sampled.

One fecal sample was collected from each eligible cow during the same clinical examination. Because the study was cross-sectional, the single fecal sample was intended to characterize the microscopic *B. coli* count at the time of sampling rather than the cows’ longer-term shedding pattern. Repeated fecal sampling was not performed; therefore, possible temporal variation in protozoal shedding could not be assessed. The sampling procedure was incorporated into the routine veterinary assessment to minimize additional handling, restraint, and animal-associated stress. Fecal material was collected either directly from the rectum or immediately following spontaneous defecation [24]. When a cow defecated spontaneously during the clinical examination, freshly voided fecal material was collected immediately using a clean disposable glove. Care was taken to collect the sample before it encountered the barn floor, bedding, manure, feed, water, or other potential sources of environmental contamination. If spontaneous defecation did not occur during the clinical examination, fecal material was obtained during a routine rectal examination. A new disposable rectal glove was used for each cow, and the sample was carefully collected.

Approximately 10 g of fecal material was collected from each cow and transferred into an individually labeled sterile 30 mL universal specimen container (Avanti Medical; FORANS Eesti AS, Tallinn, Estonia). Each container was sealed immediately after sampling and matched with the corresponding individual cow identification number. Sampling equipment and disposable gloves were replaced between animals to prevent cross-contamination.

Immediately after collection, the fecal samples were maintained at 4 °C and transported to the laboratory within 12 h. Until coprological examination, the samples remained refrigerated and were not frozen.

No cow was restrained or subjected to an additional procedure exclusively for the purposes of this study. The collection of freshly voided feces was considered non-invasive, whereas rectal sampling was regarded as a minimally invasive procedure performed as part of the routine clinical examination.

### 2.4. Coprological Examination

All fecal samples were analyzed in the Laboratory of Clinical Tests at the Large Animal Clinic, Veterinary Academy, Lithuanian University of Health Sciences, Kaunas, Lithuania. Before examination, the samples were thoroughly mixed to obtain a homogeneous distribution of parasitic stages. Each sample was examined using a sedimentation technique and a modified McMaster method. The sedimentation method was used for the microscopic detection of *B. coli*, whereas the modified McMaster method was applied to obtain a semi-quantitative estimate of its abundance.

#### 2.4.1. Sedimentation Technique

For the sedimentation procedure, 5 g of fresh feces was mixed thoroughly with 125 mL of tap water. The resulting suspension was filtered into a conical sedimentation vessel to remove coarse fecal material. The filtrate was left undisturbed for 10–30 min to allow the suspended material and parasitic stages to settle. After sedimentation, the supernatant was carefully decanted without disturbing the sediment remaining at the bottom of the vessel. The vessel was refilled with 125 mL of tap water, and the sediment was resuspended. The suspension was again left undisturbed for 10–30 min, after which the supernatant was carefully removed. Subsequently, 50 mL of tap water and three drops of methylene blue solution were added to the remaining sediment. After 5 min, the supernatant was decanted, leaving the final sediment at the bottom of the vessel. One drop of the sediment was transferred onto a clean microscope slide and covered with coverslip. The preparation was examined systematically under a light microscope at 40× magnification. Structures morphologically consistent with *B. coli* were identified based on their size, shape, and characteristic internal and external morphological features. Representative photographs illustrating the sedimentation procedure and microscopic examination of fecal samples are shown in Figure 2.

#### 2.4.2. McMaster Method

A modified McMaster method was used to obtain a semi-quantitative estimate of *B. coli* abundance in the fecal samples. For each sample, 4.0 g of fresh feces was placed into a clean container and mixed thoroughly with 50.0 mL of tap water. The suspension was left undisturbed for 30 min [25]. After this period, the suspension was mixed again and filtered through gauze into a clean container to remove coarse fecal debris. A 10.0 mL portion of the filtrate was transferred into a centrifuge tube and centrifuged for 7 min at 1200 rpm. Following centrifugation, the supernatant was carefully discarded without disturbing the sediment. The sediment was then resuspended in 4.0 mL of sodium chloride flotation solution. The resulting suspension was mixed thoroughly, and both chambers of a McMaster counting slide were filled using a pipette. Care was taken to avoid the formation of air bubbles and to ensure even chamber filling. The loaded McMaster chamber was left undisturbed for 2–3 min before microscopic examination. Both chambers were examined systematically under a light microscope. Structures morphologically consistent with *B. coli* were counted within the examined microscopic fields. The result for each sample was recorded as the number of *B. coli* structures observed per microscopic field and was used as a semi-quantitative indicator of relative abundance. No conversion to cysts per gram of feces was performed. Therefore, the recorded values represented relative microscopic counts rather than an absolute concentration of *B. coli* stages per gram of fecal material.

### 2.5. Statistical Analysis

All statistical analyses were performed using IBM SPSS Statistics for Windows, Version 30.0 (IBM Corp., Armonk, NY, USA). Prior to hypothesis testing, the distributional characteristics of all continuous variables were evaluated using descriptive statistics, histograms, Q–Q plots, and the Shapiro–Wilk normality test. The distribution of *B. coli* counts was highly skewed, with a large proportion of low or zero values and several markedly high observations. Because no established thresholds or clinical cut-offs exist in the literature for classifying *B. coli* infection intensity in cattle, an unsupervised data-driven approach was applied to define biologically meaningful groups.

To identify natural clusters within the *B. coli* count data, a Two-Step Cluster Analysis was performed using log-likelihood as the distance measure and Schwarz’s Bayesian Information Criterion (BIC) for optimal cluster number selection. This algorithm is well suited for continuous, non-normally distributed data with potential outliers, and automatically determines the most parsimonious grouping structure. The analysis identified two distinct clusters, characterized by clearly separated centroid means. Cattle in Cluster 1 exhibited markedly higher Balantidium counts (mean ≈ 43.4), whereas cattle in Cluster 2 showed low infection levels (mean ≈ 8.4). The silhouette measure of cohesion and separation indicated good overall cluster quality. The resulting cluster membership variable was saved and used as the categorical grouping factor for all subsequent comparisons.

After cluster formation, the effect of *B. coli* infection level on physiological and production traits was evaluated. The following dependent variables were analyzed: rumination time (min/day), daily milk yield (kg), milk fat (%), protein (%), lactose (%), and SCC. Because most variables exhibited non-normal distributions, non-parametric Mann–Whitney U tests were used to compare medians between the two Balantidium clusters. For variables that met assumptions of normality and homogeneity of variance, independent-samples *t*-tests were applied. Where appropriate, effect sizes were reported to facilitate interpretation of biological relevance.

In cases where adjustment for potential confounders was required (e.g., body condition score, DIM, or lactation number), General Linear Models (GLMs) with cluster membership as a fixed factor and relevant covariates were fitted. Estimated marginal means and pairwise comparisons were obtained using Bonferroni correction.

In addition to the cluster-based comparisons, zero-order Pearson correlation coefficients were calculated to assess the associations between microscopic *B. coli* counts and total eating time, rumination time, daily milk yield, milk fat-to-protein ratio, lactose concentration and log10-transformed SCC. Partial correlation analyses were subsequently performed to assess the associations while controlling for parity and DIM. All tests were two-tailed, and statistical significance was set at *p* < 0.05.

## 3. Results

### 3.1. Distribution of B. coli Counts and Cluster Formation

Microscopic *B. coli* counts showed a markedly right-skewed distribution, with most cows exhibiting relatively low counts and a smaller number of cows showing substantially higher values. As no established clinical or biological thresholds were available for categorizing *B. coli* counts in dairy cattle, a Two-Step Cluster Analysis was performed.

The analysis identified two clusters. The higher-count cluster comprised 12 cows and had a mean microscopic *B. coli* count of approximately 43.4, whereas the lower-count cluster comprised 71 cows and had a mean count of approximately 8.4. The silhouette measure indicated good overall cluster quality. Cluster membership was subsequently used as the grouping variable for comparisons of the investigated traits.

### 3.2. Comparison of Investigated Traits Between B. coli Count Clusters

No statistically significant differences were detected between the higher- and lower-count clusters for rumination time, DIM, milk fat-to-protein ratio, lactose concentration, or log10-transformed somatic cell count. Median rumination time was 225.0 min/day in both clusters. The interquartile range was 168.0–281.0 min/day in the higher-count cluster and 222.0–283.0 min/day in the lower-count cluster. The difference was not statistically significant (U = 399.0, Z = −0.350, *p* = 0.727). Median daily milk yield was 49.0 kg/day in both clusters, with interquartile ranges of 44.0–55.1 kg/day in the higher-count cluster and 43.6–55.0 kg/day in the lower-count cluster (U = 397.5, Z = −0.367, *p* = 0.712). The median milk fat-to-protein ratio was 1.14 in both clusters, with an interquartile range of 1.02–1.32. No statistically significant difference was observed (U = 396.0, Z = −0.265, *p* = 0.791). Median lactose concentration was 4.57% in both clusters, with an interquartile range of 4.57–4.63%. The difference was not statistically significant (U = 246.0, Z = −0.223, *p* = 0.823). Median log10-transformed SCC was 1.60 in both clusters. The interquartile range was 1.57–1.79 in the higher-count cluster and 1.50–1.79 in the lower-count cluster. No statistically significant difference was detected (U = 387.0, Z = −0.409, *p* = 0.683; Table 3). 

### 3.3. Zero-Order Correlations Between B. coli Counts and Investigated Traits

The zero-order correlations between microscopic *B. coli* counts and the investigated traits were weak and positive. Correlation coefficients were 0.117 for total eating time, 0.072 for rumination time, 0.097 for daily milk yield, 0.091 for the milk fat-to-protein ratio, 0.190 for lactose concentration, and 0.027 for log10-transformed somatic cell count.

The strongest zero-order association was observed between microscopic *B. coli* counts and lactose concentration, although the correlation remained weak. The weakest association was observed for log10-transformed somatic cell count. None of the zero-order correlations reached statistical significance (Figure 3).

### 3.4. Partial Correlations Adjusted for Parity and Days in Milk

After adjustment for parity and days in milk, the partial correlations between microscopic *B. coli* counts and total eating time (r = 0.105), rumination time (r = 0.036), daily milk yield (r = 0.060), milk fat-to-protein ratio (r = 0.064), and log10-transformed somatic cell count (r = 0.015) remained weak and statistically non-significant.

A positive partial correlation was observed between microscopic *B. coli* counts and milk lactose concentration (r = 0.296, *p* = 0.007). Although this was the only statistically significant adjusted association, its magnitude remained small (Figure 4).

## 4. Discussion

*B. coli* is detected in the gastrointestinal tract of livestock, often as an asymptomatic carrier state, yet its biological significance in clinically healthy dairy cows remains poorly understood [26]. The available research is particularly lacking in information regarding the potential correlation between naturally fluctuating fecal counts of this protozoan and precision dairy-farming parameters, including automated behavioral monitoring, milk production, milk composition, and udder health status [6,26,27,28]. The present study detected no statistically significant differences in rumination time, milk yield, milk fat-to-protein ratio, lactose concentration or log-transformed SCC between cluster of cows with higher versus lower microscopic *B. coli* counts. Most zero-order and adjusted correlations (controlling for parity and DIM) were also weak. The only statistically significant association identified was between microscopic *B. coli* counts and milk lactose concentration after adjustment for parity and DIM. Therefore, the study objective was achieved by determining whether microscopic *B. coli* counts were associated with the investigated behavioral, production and milk-composition indicators. The hypothesis was not broadly supported, because most associations were weak and statistically non-significant, although an adjusted association with milk lactose concentration was identified.

Previous coprological studies have demonstrated that cattle can harbor and shed *B. coli* cysts or trophozoites, although the clinical status and production consequences of positive animals have not always been evaluated in detail [29,30]. Therefore, detection of *B. coli* in feces should not by itself be interpreted as evidence of clinical disease, as infection may remain subclinical, and its clinical expression varies among hosts. The development of clinical disease is hypothesized to be influenced by a range of host-related factors, including intestinal mucosal integrity, immune competence, nutritional condition and concurrent disease [7,26]. This suggests that these factors may be more significant than protozoan abundance alone in determining the onset of clinical disease.

It is important to note that environmental and herd management conditions have the potential to influence exposure and transmission, thereby contributing to variations in prevalence among cattle populations [30]. The present study included only clinically healthy cows within a restricted range of parity and DIM, resulting in a relatively homogeneous population with respect to major physiological and production-related factors. This restricted eligibility may have reduced between animal variability and may partly explain the absence of detectable behavioral or production differences between cows with higher and lower microscopic *B. coli* counts.

Rumination time is widely known as a sensitive, although non-specific, behavioral indicator of dairy cow health, whereas changes in eating time may provide additional information on altered physiological or health status [31,32]. Reduced rumination and eating activity may reflect lower feed intake or altered rumen function and have been observed in dairy cows affected by metabolic, digestive, and other health disorders [16,32,33]. Rumination and feeding behavior are widely used as indicators of changes in the health of dairy cows. This is due to the fact that metabolic and digestive disorders are frequently accompanied by reductions or alterations in these activities [16,34,35]. The absence of clear associations with rumination and eating behavior in the present study suggests that natural variation in microscopic *B. coli* counts was not accompanied by detectable alterations in these behavioral indicators under the management conditions of the investigated herds. However, it is important to note that these findings should not be interpreted as evidence that subtle gastrointestinal or physiological alterations were entirely absent, because digestive function and individual dry-matter intake were not assessed directly. Furthermore, each cow was represented by a single fecal sample, which reflected the microscopic *B. coli* count at one sampling point rather than its longer-term shedding pattern. Consequently, there is a necessity for longitudinal studies that combine repeated fecal sampling with closely aligned sensor-derived behavioral data. This will allow for the determination of whether short-term variation in *B. coli* counts coincides with transient changes in rumination or feeding behavior.

Milk-yield reduction is one of the major sources of economic loss associated with common subclinical disorders in dairy cows, particularly subclinical mastitis and ketosis [36,37,38]. Gastrointestinal nematode infections are a significant factor in reducing productive performance, with the resulting effects of decreased feed intake, impaired digestion and reduced nutrient utilization. The diversion of nutrients and energy toward immune responses and the repair of damaged gastrointestinal tissues is also a key factor in the reduction in productivity [39,40,41,42]. Variation in microscopic *B. coli* counts was not associated with measurable differences in milk production in the clinically healthy cows examined under the conditions of the studied herd. In a previous study of Red Chittagong cattle with subclinical gastrointestinal parasitosis, Rahman and Samad [28] reported an average increase in milk yield of 0.32 L/cow/day following antiparasitic treatment. The same study reported a 42.85% reduction in *B. coli* trophozoite counts; however, the animals harbored mixed gastrointestinal parasitic infections, and the observed increase in milk production could not be attributed specifically to *B. coli*. Differences in breed, baseline milk yield, parasitic composition, treatment-based study design, and herd conditions further limit direct comparison with the present findings. Consequently, the absence of a detectable association in the current study should not be interpreted as evidence that *B. coli* can never have production-related relevance, particularly in cows with clinically apparent gastrointestinal disease, concurrent infections, or substantial systemic compromise.

The milk fat-to-protein ratio is widely utilized as a non-invasive, indirect indicator of the energy and metabolic status of dairy cows, particularly during the early stages of lactation. It has been established that elevated milk fat-to-protein ratio values are indicative of a more negative energy balance, augmented mobilization of body reserves, and an elevated risk of ketosis [21,43]. In contrast, reduced milk fat-to-protein ratio values may be observed in bovines with modified rumen fermentation or subacute ruminal acidosis. Nevertheless, it is imperative to interpret milk fat-to-protein ratio as a screening indicator rather than a standalone diagnostic measure [44]. The absence of a clear association with the milk fat-to-protein ratio suggests that variation in microscopic *B. coli* counts was not accompanied by a detectable change in this indirect marker of metabolic status under the conditions of the studied herd. Nevertheless, because milk fat-to-protein ratio is influenced by multiple physiological and management-related factors and is not specific to gastrointestinal dysfunction or parasitic infection, the absence of an association does not exclude subtle metabolic or digestive alterations that were not directly assessed in the present study [45].

SCC is widely used as a cow-level indicator of mammary inflammation and as a screening measure for subclinical intramammary infection in dairy cattle [23,46]. A healthy mammary gland generally exhibits relatively low levels of SCC, while elevated SCC levels are more frequently associated with the presence of mastitis pathogens and an inflammatory cellular response within the udder [46]. In contrast, *B. coli* is an enteric protozoan whose principal site of colonization is the large intestine; consequently, a direct biological relationship between fecal *B. coli* counts and mammary inflammation would not necessarily be expected [9]. The absence of a detectable association with SCC in the present study suggests that variation in microscopic *B. coli* counts was not accompanied by measurable changes in mammary inflammatory status in the clinically healthy cows examined. Previous studies have shown that SCC is driven primarily by intramammary infection and mastitis-related factors rather than by enteric parasitism. Williamson et al. [46] reported a clear association between elevated SCC and bacteriological evidence of intramammary infection, supporting the interpretation of SCC as an udder-specific inflammatory indicator. Therefore, it can be concluded that the absence of a relationship with SCC in the present study should be interpreted specifically as a lack of evidence for an association between microscopic *B. coli* counts and udder inflammatory status, rather than proof that no systemic or gastrointestinal effects were present.

The most notable finding of the present study was the positive association between microscopic *B. coli* counts and milk lactose concentration observed after adjustment for parity and DIM. Lactose is the osmotically active principal constituent of bovine milk, contributing significantly to the regulation of water transfer into the mammary secretion and, consequently, milk volume [22,47]. Lactose synthesis occurs in mammary epithelial cells and depends on the supply and uptake of glucose by the mammary gland, linking lactose production to the metabolic demands of lactation [47]. Milk lactose concentration is not constant and may vary according to parity and stage of lactation, even in cows without evidence of udder inflammation [22]. Lower lactose concentrations have also been associated with mammary-quarter inflammation, elevated SCC, and indicators of negative energy balance, demonstrating that lactose reflects several physiological and health-related processes rather than a single biological pathway [22]. The positive adjusted association observed in the present study should therefore not be interpreted as evidence that *B. coli* directly stimulates lactose synthesis. The increase in the correlation coefficient after adjustment suggests that parity and days in milk influenced the crude association between the two variables; however, residual confounding by unmeasured nutritional, metabolic, or cow-level factors cannot be excluded. Moreover, no corresponding association was observed with milk yield, the milk fat-to-protein ratio, or log10-transformed SCC, providing no consistent pattern that would support a clear physiological mechanism linking microscopic *B. coli* counts with mammary lactose synthesis. Because several behavioral, production, and milk-composition outcomes were examined, the possibility of a chance finding related to multiple statistical comparisons should also be considered. Accordingly, the association with lactose should be regarded as exploratory and hypothesis-generating until it is confirmed in a larger, independent population using repeated sampling and more comprehensive metabolic and nutritional measurements.

The main strength of this study was the use of a relatively homogeneous group of clinically healthy Holstein cows maintained under the same commercial farm conditions, with behavioral and production data obtained from automated monitoring systems. Several limitations should nevertheless be acknowledged. The study was conducted in a single herd and included only 83 cows, with 12 animals assigned to the higher-count cluster, which may have limited the ability to detect small between-group differences and restricts the generalizability of the findings. Only one fecal sample was collected from each cow, and therefore the microscopic count reflected a single sampling point rather than a longer-term shedding pattern. In addition, *B. coli* abundance was expressed as a microscopic count per field rather than as a standardized number of cysts per gram of feces, and species identification was based on morphology without molecular confirmation. Consequently, the results should be interpreted as relative microscopic abundance of structures consistent with *B. coli*, rather than as an absolute or molecularly confirmed parasite burden. Finally, the cross-sectional design does not allow causal inference, and potentially relevant factors such as individual dry-matter intake, gastrointestinal function, gut microbiota, and systemic inflammatory or metabolic biomarkers were not assessed. These limitations may have reduced the ability to detect subtle associations between microscopic *B. coli* counts and the investigated behavioral and milk-composition indicators. Therefore, although the study objective was addressed, the ability to fully test the proposed hypothesis was constrained by the cross-sectional design, single-time-point sampling, limited sample size and the absence of molecular confirmation and additional physiological measurements. Furthermore, because this study was conducted in a single high-producing commercial herd using automated milking and monitoring systems, the findings may not be directly generalizable to farms with different productivity levels, management practices, housing conditions, feeding strategies or animal health status.

Future studies should include larger populations from multiple herds representing different levels of productivity and management intensity and should use repeated fecal sampling to account for temporal variation in protozoal shedding. Standardized quantitative methods, such as reporting cyst counts per gram of feces, would improve comparability between animals and studies. Molecular confirmation should be incorporated to verify species identity and characterize potential genetic variation among *B. coli* isolates. Longitudinal designs would help determine whether within-cow changes in microscopic *B. coli* abundance precede changes in behavior, milk production, or milk composition. Concurrent assessment of individual feed intake, gut microbial composition, and metabolic and inflammatory biomarkers may further clarify whether the observed association with milk lactose reflects an indirect relationship involving host nutrition, intestinal ecology, or physiological status.

## 5. Conclusions

The present study provides evidence that microscopic *B. coli* counts have limited value as standalone indicators of behavioral, production or udder-health alterations in clinically healthy cows. Zero-order correlations between microscopic *B. coli* counts and the evaluated indicators were weak and statistically non-significant (r = 0.027–0.190). After adjustment for parity and DIM, only milk lactose concentration showed a statistically significant association (partial r = 0.296, *p* = 0.007), whereas the remaining adjusted correlations were weak or negligible (r = 0.015–0.105). Given the absence of a consistent pattern across the other evaluated indicators, the cross-sectional design, and the number of statistical comparisons performed, this result should be regarded as exploratory and hypothesis-generating rather than as evidence of a direct biological effect of *B. coli*. Overall, the findings indicate that higher microscopic *B. coli* counts were not consistently associated with measurable alterations in the evaluated behavioral, production, or udder-health indicators in clinically healthy cows. Confirmation in larger, longitudinal, multi-herd studies using repeated sampling and molecular identification is warranted.

## Figures and Tables

**Figure 1:**
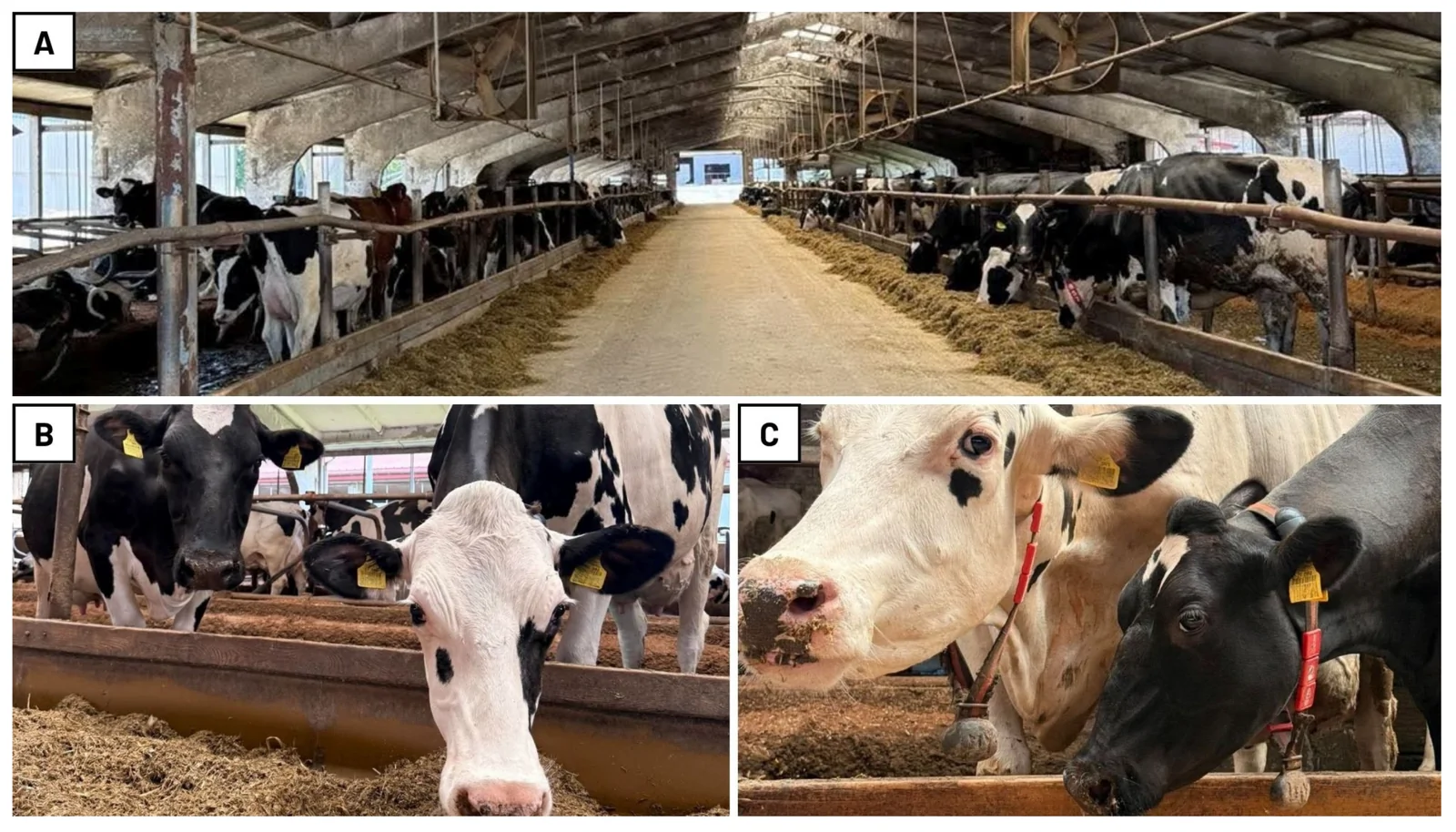
Representative photographs of the investigated commercial dairy farm: (**A**) general view of the free-stall housing and feeding alley; (**B**) Holstein dairy cows at the feeding area under the same management conditions; and (**C**) cows equipped with Lely Qwes-H/HR neck-mounted sensors used for automated behavioral monitoring.

**Figure 2:**
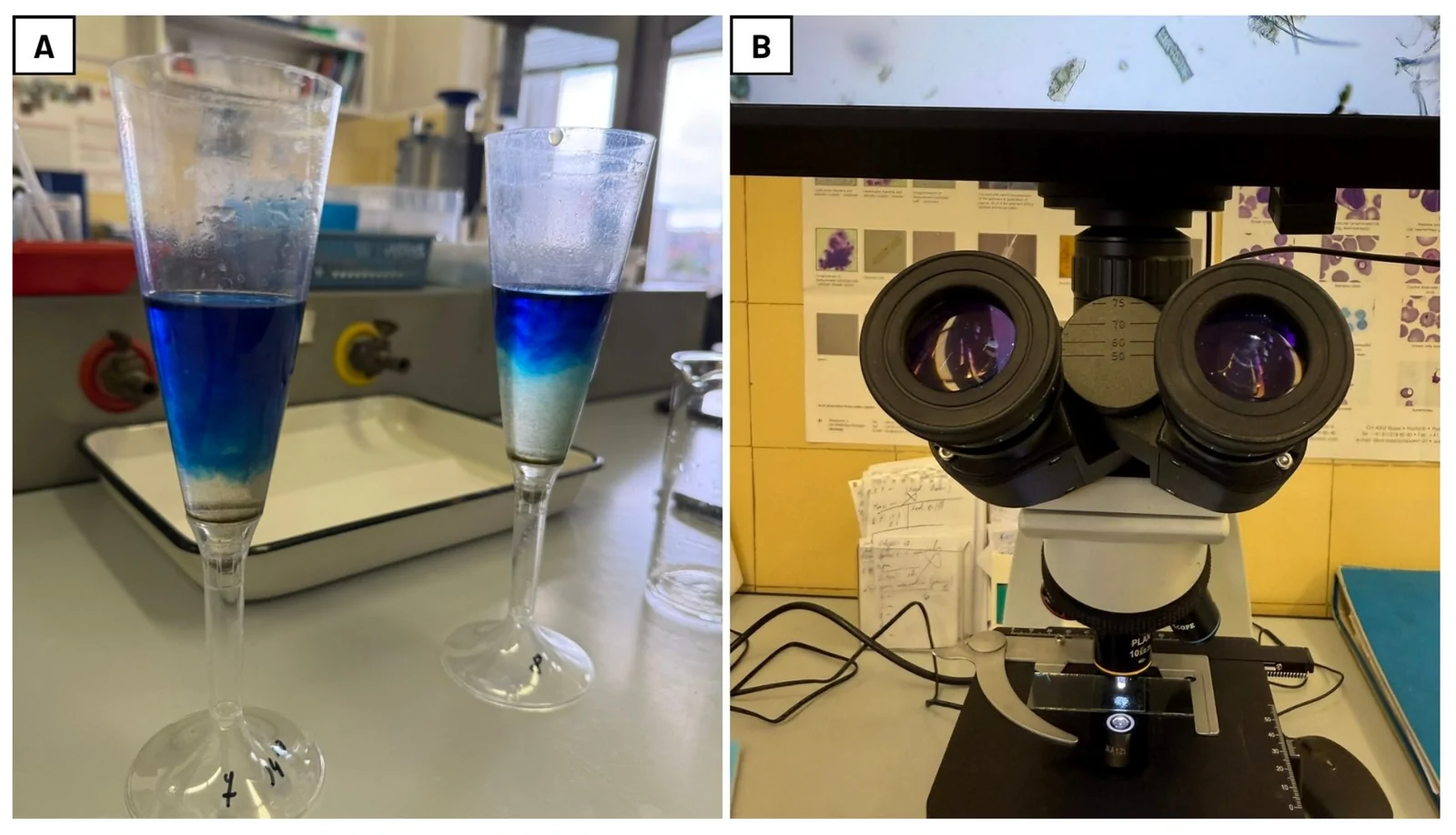
Representative photographs of the laboratory procedures used for coprological examination: (**A**) sedimentation of fecal samples during sample preparation; (**B**) microscopic examination of the prepared fecal samples.

**Figure 3:**
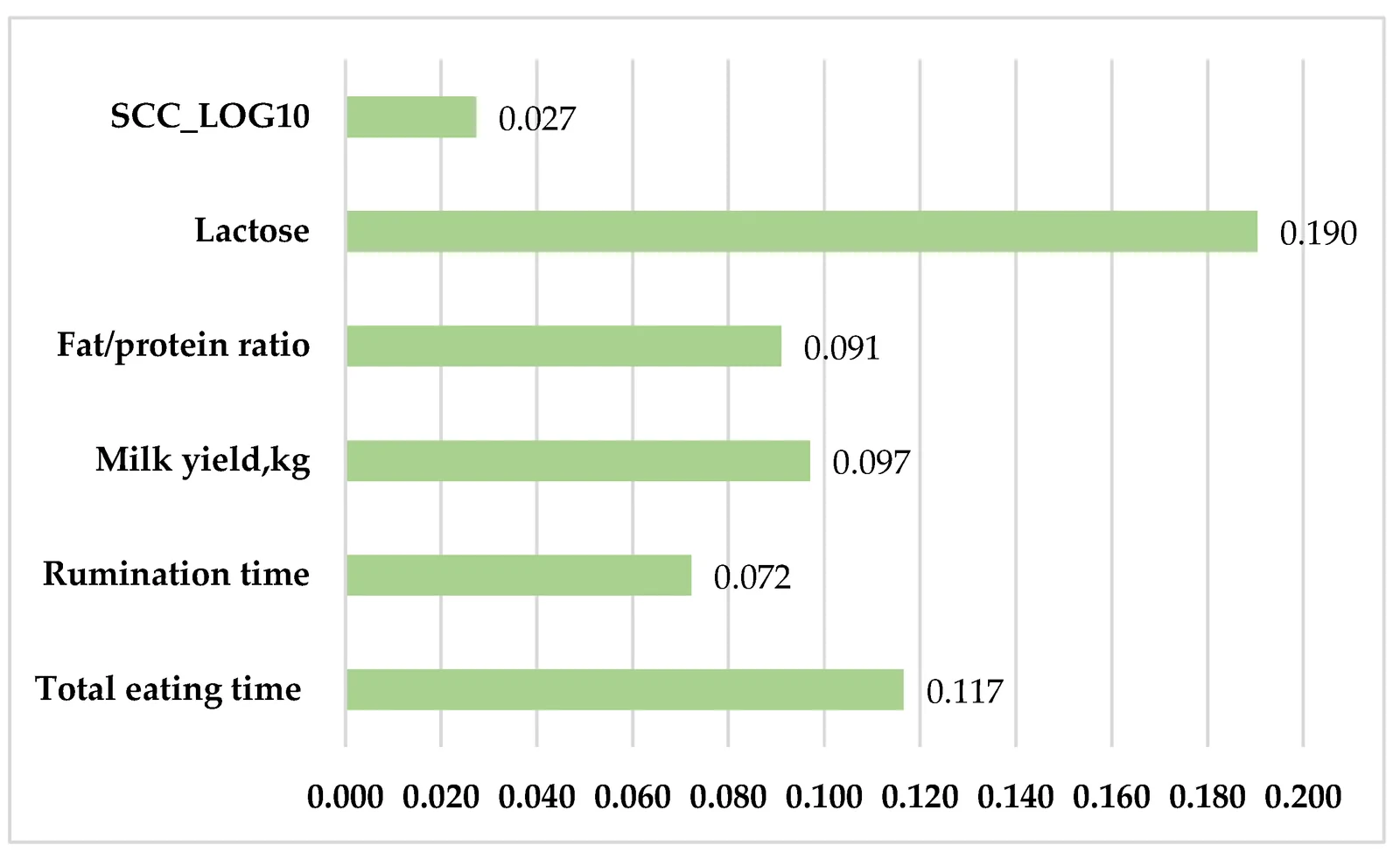
Zero-order (Pearson) correlations between microscopic *B. coli* counts and the investigated traits.

**Figure 4:**
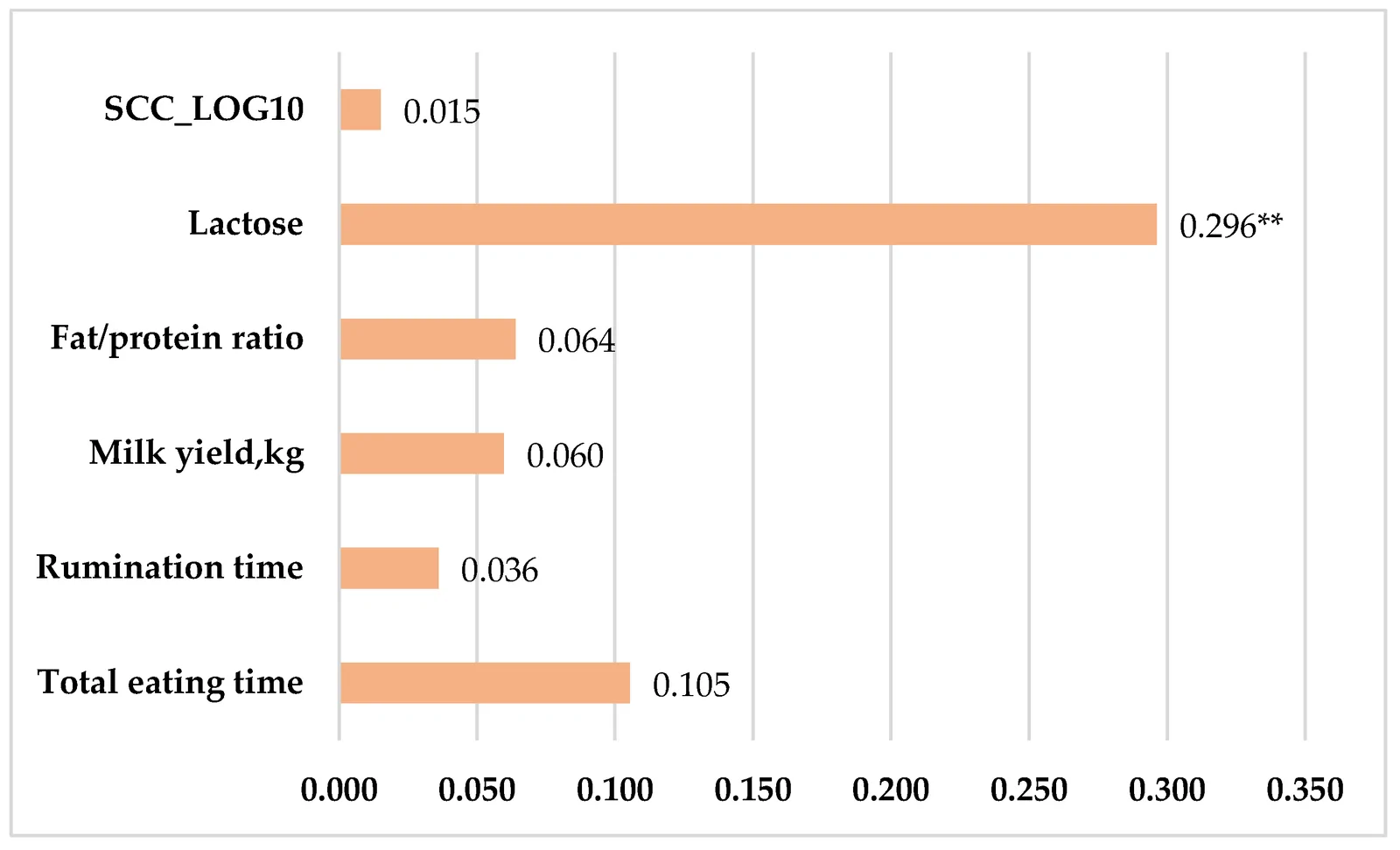
Zero-order (Pearson) and partial correlations between Balantidium count and selected traits. Partial correlations adjust for parity and days in milk. Significance: ** *p* < 0.01.

**Table 1:** Composition of the TMR fed to the study cows.

TMR Component	Value
Corn silage	30%
Grass hay	4%
Grass silage	10%
Grain concentrate mash	50%
Mineral mix	6%

TMR—total mixed ratio.

**Table 2:** Chemical composition of TMR.

TMR Component	Value
Dry Matter (DM)	47.8%
Neutral Detergent Fiber (NDF)	29% of DM
Acid Detergent Fiber (ADF)	17.5% of DM
Non-Fiber Carbohydrates (NFC)	38.6% of DM
Crude Protein (CP)	15.8% of DM

**Table 3:** Comparison of physiological and milk-production traits between *Balantidium* infection clusters (Mann–Whitney U tests). *n*—number of cows in each group; IQR—interquartile range; U—Mann–Whitney U test statistic; Z—standardized test statistic; *p*-value—statistical significance level. Cluster 1—high infection; Cluster 2—low infection.

Trait	Cluster	*n*	Median	IQR	U	Z	*p*-Value
Rumination time (min/day)	High infection	12	225.0	168.0–281.0	399.0	−0.350	0.727
	Low infection	71	225.0	222.0–283.0			
Daily milk yield (kg/day)	High infection	12	49.0	44.0–55.1	397.5	−0.367	0.712
	Low infection	71	49.0	43.6–55.0			
Milk fat/protein ratio (%)	High infection	12	1.14	1.02–1.32	396.0	−0.265	0.791
	Low infection	71	1.14	1.02–1.32			
Lactose (%)	High infection	12	4.57	4.57–4.63	246.0	−0.223	0.823
	Low infection	71	4.57	4.57–4.63			
Somatic cell count (log10 SCC)	High infection	12	1.60	1.57–1.79	387.0	−0.409	0.683
	Low infection	71	1.60	1.50–1.79			

## Data Availability

The original contributions presented in this study are included in the article. Further inquiries can be directed to the corresponding author.

## Abbreviations

The following abbreviations are used in this manuscript:

SCC	Somatic cell count
DIM	Days in milk
TMR	Total mixed ration
DM	Dry matter
NDF	Neutral detergent fiber
ADF	Acid detergent fiber
NFC	Non-fiber carbohydrates
CP	Crude protein
BIC	Schwarz’s Bayesian Information Criterion
GLM	General Linear Model
